# Age heterogeneities in child growth and its associated socio-demographic factors: a cross-sectional study in India

**DOI:** 10.1186/s12887-022-03415-x

**Published:** 2022-06-30

**Authors:** Suryakant Yadav, Pravat Bhandari

**Affiliations:** 1 Department of Bio-Statistics and Epidemiology, International Institute for Population Sciences, Mumbai, 400088 India; 2 International Institute for Population Sciences, Mumbai, 400088 India

**Keywords:** Age heterogeneities, Child growth, Height-for-age, Weight-for-age, Socio-demographic factors, Multilevel modelling, India

## Abstract

**Background:**

The impacts of socio-demographic and environmental risk factors on child growth have been widely documented. However, it remains unclear whether the impacts of such risk factors on child growth have remained static or changed with child’s age. The present study aims to assess the underlying age heterogeneities in child growth and its potential determinants over age in under-five children.

**Methods:**

Cross-sectional data on child height (measured as height-for-age z-score, i.e., HAZ) and weight (measured as weight-for-age z-score, i.e., WAZ) and potential confounding factors from India’s 2015–16 National Family Health Survey (NFHS) were used to construct anthropometric age-profiles by a number of bio-demographic and socioeconomic characteristics. Further, age-interacted multilevel regression analyses were performed to examine differential effects of such/those risk factors on child height and weight by age.

**Results:**

Faltered height and weight growth during first two years of life was noticed in children of all socioeconomic groups studied, albeit with varying magnitude. In case of child’s height, factors such as short birth interval, higher birth order, maternal education, household wealth, district level mortality rate have shown strong interaction with child’s age during the first 23 months, signifying their age-varying role in different developmental stages of child growth. These factors explain the observed upward and downward shifts in height curve during first two years. Some of these variables (e.g., household wealth) have shown even stronger age interactions after the second birthday of children. For child’s weight, interactive effects of most socio-demographic risk factors attenuated parabolically with child’s age.

**Conclusions:**

The impacts of several risk factors, measured at the child, mother, community, and district levels, on child growth indicators varied significantly with the child’s age. Nutritional interventions aimed at preventing poor linear growth in children in India should consider these underlying age heterogeneities for growth determinants into account.

**Supplementary Information:**

The online version contains supplementary material available at 10.1186/s12887-022-03415-x.

## Background

### Introduction

Linear growth faltering (also known as linear growth retardation), defined as slow rate of gain in height or weight relative to one’s age, is a direct indicator of poor nutritional status among children. Cross-country analyses suggest that growth faltering is apparent among children in many low- and middle-income countries; however, its magnitude in a country is likely to depend on the phase of economic as well as demographic development [1–3]. Numerous studies suggest that growth faltering in children does not occur uniformly over age and time: the magnitude of faltering tend to vary with child’s age and socio-economic status of households [2–4]. In many developing countries, initially average height-for-age (HAZ) and weight-for-age (WAZ) are close to the international standard at birth, but they decline sharply with age, resulting in a downward shift in postnatal years and thus affects the entire growth curve of the children [1, 5].

While the downward shift because of growth faltering is conspicuous during the first 2 years of postnatal years, its detrimental consequences follow throughout childhood. Pieces of literature suggests that child’s growth retardation is associated with short stature, impaired cognition and reduced economic productivity while in adulthood [6–14]. In addition to that, children experiencing faltered growth are at much higher risk of developing cardiovascular disease and nutrition-related disorders (e.g., diabetes and obesity) in later stages of their life [15]. Recognizing the heavy economic cost associated with early childhood growth faltering, the global nutrition community has recommended scaling up nutritional interventions during the early years of life so that a large proportion of children could be prevented from the scourge of undernutrition [1, 16].

In India, the elimination of child undernutrition has been a key public health challenge. Nutrition-specific and nutrition-sensitive interventions in place have shown unsatisfactory improvements in terms of prevalence of undernutrition. In particular, stunting declined from 52% in 1992–93 to 38% in 2015–16; however, this high prevalence of stunting among Indian children is ranked ‘on course’ in the global nutrition index [17]. The ‘on course’ status of stunting (measured by height-for-age z-score (HAZ)) and underweight (measured by weight-for-age z score (WAZ)) has attracted tremendous attention among the researchers in the last two decades, yielding a vast literature empirically assessing, re-assessing, and debating the relative roles of bio-demographic, socioeconomic, and environmental determinants on nutritional outcomes [18–22]. A study by Mamidi et al. [23] examined the age patterns of growth faltering in children of India wherein data was limited between two rounds of NFHS data. Nevertheless, very little has been explored regarding the age patterns of growth faltering in children in India. A scrutiny of the age patterns of child growth curve is crucial for developing country India because it would facilitate the interventions emerged out of policy formulations and programme implementations aimed at preventing undernutrition in children. The association of child’s growth curve with socio-economic factors varies as a function of the child’s age that need to accounted for the interventions will have the maximum impact. While there are evidences of the socio-economic and environmental determinants of child’s growth in terms of stunting and underweight, the consequences of age heterogeneity on the determinants of child’s growth are barely scrutinised. In view of nuances of child’s growth curve, in this research work, we study age patterns in growth faltering in Indian children to assess the underlying age heterogeneities in the determinants of child growth.

In the following two sections (Sects. 1.2 and 1.3), we present a brief review of relevant literature on (1) age patterns of growth faltering and (2) age heterogeneities in child growth determinants.

### Age patterns of growth faltering

A pioneering study by Shrimpton et al. [5] analysed the worldwide patterns of growth faltering in under-five children, using 1976 NCHS growth reference. Their findings suggest that the HAZ of young children in developing countries declines sharply from birth to around two years of age. Further, Victora et al. [1], using 2006 WHO growth standard, observed strikingly similar patterns of growth faltering to those reported by Shrimpton et al. A recent multi-country analysis by Rieger and Trommlerová [3] shows that the magnitude of height-for-age decline, particularly between birth and two years of age, differs significantly across the regions of developing countries: declines are more pronounced for Sub-Saharan Africa and South-East Asia as compared to other developing regions such as North Africa, Latin America, and Central Asia. Maleta et al. [24] and Nabwera et al. [25] using longitudinal anthropometric data of Malawian and Gambian children, respectively show that faltering in height growth occurs during first 24 to 36 months.

**Fig. 1:**
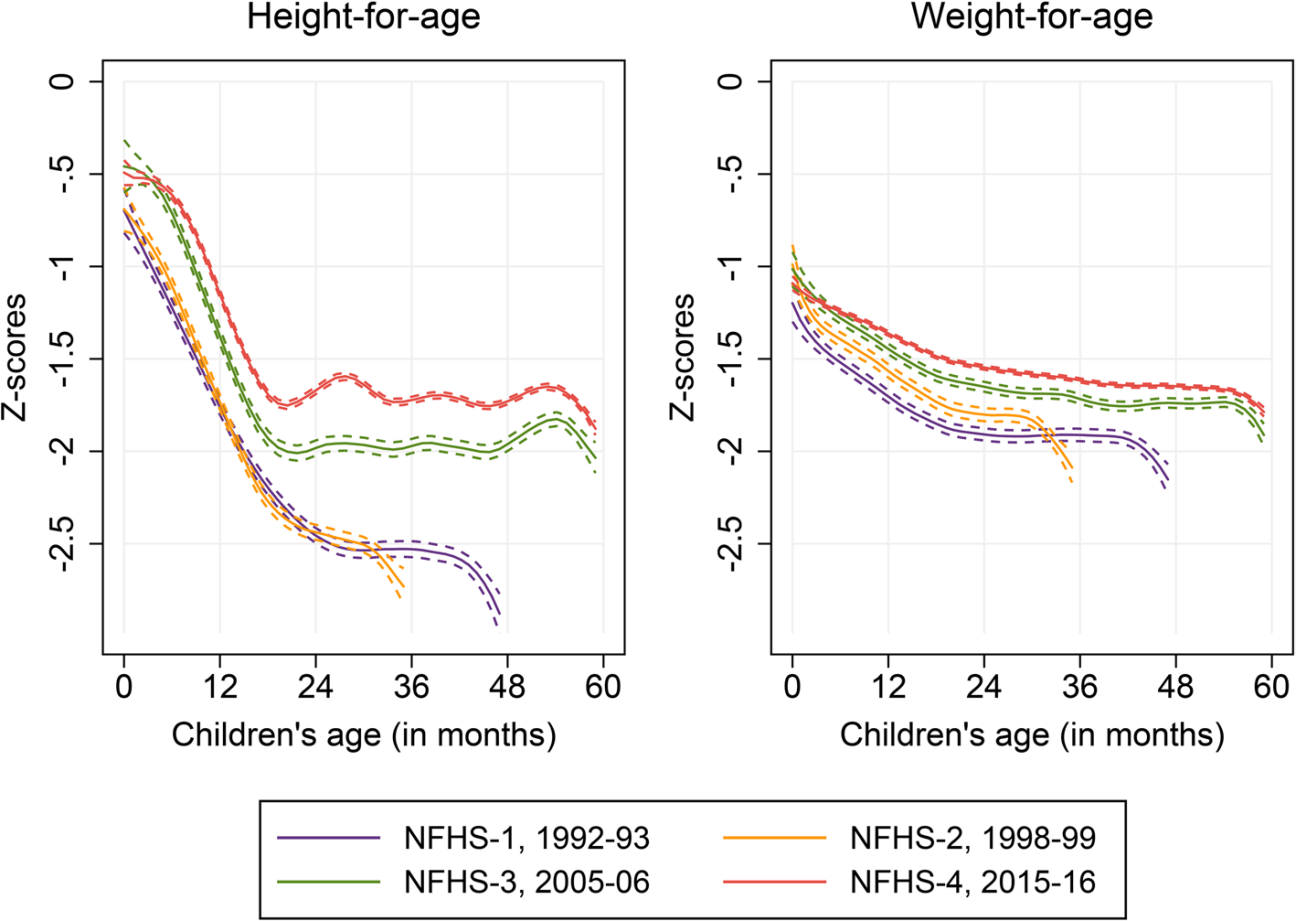
Growth faltering pattern in height (HAZ) and weight (WAZ) in under-five children, India, 1992–93—2015–16

NFHS data show that on average newborns in India begin their postnatal life with a quantum of stunting and underweight, i.e., (mean) HAZ and WAZ below the WHO 2006 Child Growth Standard (see Fig. 1). Although HAZ and WAZ in infants at birth (0 month) improved between 1992–93 and 2015–16, they still cannot retain their HAZ and WAZ as they grow up to what they were born with. The HAZ values declines in the age range of 0 to approximately 23 months, and thereafter, it becomes reasonably flat. On the other hand, the decline in the WAZ values were throughout the first five years of life, albeit the decline was less steeper than that for HAZ in the age range of 0–18 months and with a reduced rate of decline in later child’s age shows a quadratic pattern.

### Age heterogeneities in child growth determinants

Researchers have increasingly identified that the effects of various socio-economic and environmental determinants on child health are not uniform across all ages rather, the effects of such determinants are likely to differ across child’s ages. For example, in two seminal studies, Case et al. [26] and Curie and Stabile [27] have found that the effect of household wealth (or income) are much stronger among older children in the USA and Canada. A similar positive effect of wealth gradient with age for child nutrition was established in a recent study for low- and middle-income countries [28]. Furthermore, similar to household wealth, the positive effect of maternal educational attainment on child health has been reported to get stronger with age [29]. In terms of environmental determinants, age-varying effects of household living conditions and household amenities on child health have been well documented. In addition to these factors, the age heterogeneities, however, believed to stem from several biological and behavioural processes [30].

The underlying mechanism through which the gap in child’s health narrows with age is the cumulative advantages in health. The cumulative advantages in health accrues due to accumulation in health capital [31–33]; such as children with better socioeconomic status, i.e., with greater household wealth, have more prolific spending for comfort living, greater consumption of rich macro- and micro-nutrients foods, and better access to preventive and curative quality healthcare services and resilience to disease, shocks, and poverty and such benefits are likely to accumulate in good health with increase in age. This cumulative advantage has been the reinforcing factor for the rich-poor divide in developing country India.

## Methods

### Data source

This paper uses data from the fourth round of the National Family Health Survey (NFHS-4), conducted in India during 2015–16. NFHS is a cross-sectional household survey, carried out at a regular interval as part of the global Demographic and Health Surveys (DHS) Program. It provides information on maternal and child health, nutrition, household demographics, socioeconomic status, and other health indicators based on nationally-representative samples. In NFHS-4, a two-stage stratified random sampling design was adopted to draw separate rural and urban samples from each of India’s 640 districts covering all 36 States and Union Territories (UTs) of India. At the first stage, 28,586 primary sampling units (PSUs), which are villages in the rural areas and census enumeration blocks (CEBs) in the urban areas, were selected through a probability proportional to size (PPS) sampling scheme. Note that, these villages in rural areas and CEBs in urban areas are collectively referred to as communities in the current paper. At the second stage, a fixed number of 22 households were sampled from each of the selected PSUs, using systematic random sampling [34].

In the sampled households, all women aged 15–49 years, irrespective of their marital status, were invited to participate in the survey. All participating women as well as their children under 5 years were eligible for height and weight biomarker measurements. Thus, the NFHS-4 dataset contains height and weight biomarker data of 236,455 children. We dropped 11,952 (5.0%) children as they are not the usual residents and dropped 10,875 (4.6%) children due to their implausible height or weight data (i.e., HAZ and WAZ score below -6 or above + 6 SD) or invalid age data. 10,095 (4.2%) children could not analysed because of missing values of covariates. Our final analytical sample included 203,533 children (aged between 0–59 months), born to 157,379 mothers, from 27,980 communities (villages and CEBs), from all 640 districts and 36 States and Union Territories of India.

### Variables

#### Outcome variable

We analysed two standard anthropometric indices, namely, height-for-age z-score (HAZ), i.e., stunting, and weight-for-age z-score (WAZ), i.e., underweight, as indicators of the growth status in Indian children. These z-scores were estimated using NFHS-4 unit level data from kid’s file against 2006 WHO child growth standards [35].

#### Explanatory variables

On the basis of the works of literature [18, 21, 22, 36–41], we analysed the potential explanatory variables for the present study at five different levels: child, mother, household, community, and district.

Child attributes that were considered as potential determinants of child growth includes sex, birth order, birth size, and preceding birth interval. Maternal attributes considered were age at marriage, educational attainment, height, body mass index (BMI), and exposure to media (i.e., TV, radio, and newspaper). Household attributes considered were use of solid cooking fuel and socioeconomic status (SES). In order to capture the socio-economic status (SES) of households, we constructed wealth index using a principal component analysis based on indicators of household assets and amenities, as suggested by Filmer and Pritchett [42]. Further, the proportion of children (12–23 months) who received essential vaccination, and the level of under-five mortality rate were considered as district attributes. Type of residence (rural and urban), the proportion of household practicing open defecation, the proportion of the poor households, and the proportion of mothers who did not attend primary schooling were considered as community attributes. Descriptive analysis for each covariates considered in this study is shown in Table 1.

**Table 1:** Descriptive statistics of sample children aged 0–59 months (*n* = 203,533)

Variable	Mean	SE
***Dependent variable***		
Height-for-age z-score	-1.483	0.004
Weight-for-age z-score	-1.521	0.003
***Independent variable***		
Child’s age (in months)	30.249	0.037
Mother’s age at marriage	18.890	0.008
Mother’s height (in cm)	151.757	0.013
Mother’s BMI (in kg/m^2^)	21.208	0.008
% Poor household in the community	50.754	0.074
% Household practices open defecation in the community	43.955	0.079
% Mother not having primary schooling in the community	31.289	0.063
% Children (6–23 months) vaccinated in the district	61.827	0.037
Rate of under-five mortality in the district (per 1000 births)	48.785	0.093
	**Weighted %**	**n**
Male	51.98	105,385
First born	37.97	74,711
Second born	32.31	63,177
Third born	15.54	33,140
Fourth or later born	14.19	32,505
Born with larger than average birth size	18.92	34,237
Born with average birth size	69.45	145,777
Born with small or very small birth size	11.63	23,519
Born with short preceding birth interval (< 24 months)	16.34	33,291
Mother completed primary schooling	13.93	29,342
Mother completed secondary schooling	46.29	93,753
Mother completed higher education	10.36	18,916
Mother has media exposure	65.46	129,109
Household uses solid cooking fuel	44.45	105,068
Richest	14.99	28,329
Rich	21.80	34,631
Middle	19.91	41,022
Poor	21.80	47,678
Poorest	24.79	51,873
Urban residence	28.30	49,156

### Statistical analysis

Our initial analysis consist of plotting the anthropometric age-profiles by a number of demographic and socioeconomic characteristics. To construct these age profiles, we first regressed anthropometric z-scores against child’s age using a kernel weighted local polynomial regression and then plotted the generated smoothed values.

The age heterogeneities that we document using the above-mentioned bivariate framework, do not account for the potential effects of biological, socioeconomic, and environmental factors on child growth. To document the underlying age heterogeneities in child growth correlates, while controlling for the impact of a number of background variables, we adopt the multilevel modelling approach, which was extended to incorporate the interactions of age-profile and relevant covariates.

For HAZ, we estimated a four-level model (Eq. 1) which takes the following form:


1


where, ${HAZ}_{imcd}$ represents height-for-age z-score of child $i$, born to mother $m$, in community $c$, and district $d$; ${\beta }_{0}$ is the constant; ${\beta }_{1}$ is a coefficient vector that captures the main relationship between HAZ and child’s age in months (main effects); ${\beta }_{2}$ is a coefficient vector for the structural break—a dummy variable that takes a value 0 for the children aged 0–23 months and takes a value 1 for the older children (i.e., 24–59 months); ${\beta }_{k}$ is a coefficient vector for ${S}_{imcd}$, which is a matrix for covariates that can be defined at the child, mother, community, and district level; *β*_*k′*_, *β*_*k″*_, and *β*_*k‴*_ capture interactions of each covariate for ${S}_{imcd}$ on child’s age, structural break, and child’s age as well as the structural break, respectively. Note that the coefficients (*β*_*k′*_) on the interaction terms between covariates and age (in continuous form) would allow us to quantify the differential effects of variables with increase of one month of child’s age throughout the first 23 months (i.e., before the structural break). While, on the other hand, the coefficients (*β*_*k″*_) on the interactions between covariates and the structural break would allow us to measure the differential effects of variables among children of older groups (24–59 months of age) [3]. The random effects ${z}_{d}$, ${v}_{cd}$, ${u}_{mcd}$, and ${\varepsilon }_{imcd},$ are the residual variances at the child, mother, community and district level, respectively. These four random effects are assumed to be independent and have a normal distribution with mean zero and variances ${\sigma }_{z}^{2}$, ${\sigma }_{v}^{2}$, ${\sigma }_{u}^{2}$ and ${\sigma }_{\varepsilon }^{2}$, respectively [43].

While our estimation strategy for WAZ remained similar as it was for HAZ, we included age-squared as a variable and interacted it with independent variables because the data points suggested a quadratic fit (Eq. 2). The reason why we do not use the structural break as an interaction term in Eq. 2 is that, unlike the HAZ age profile, the WAZ age profile did not exhibit a clear breakpoint in the curve, rather it indicated a slow rate of non-linear decrease in WAZ with children’s age. The interaction term between age and covariates captures the linear effects of age, whereas that of between age-squared and variables captures the quadratic effects of age. Thus, the four-level model estimated for WAZ takes the following form:


2


## Results

### Results from descriptive findings

**Fig. 2:**
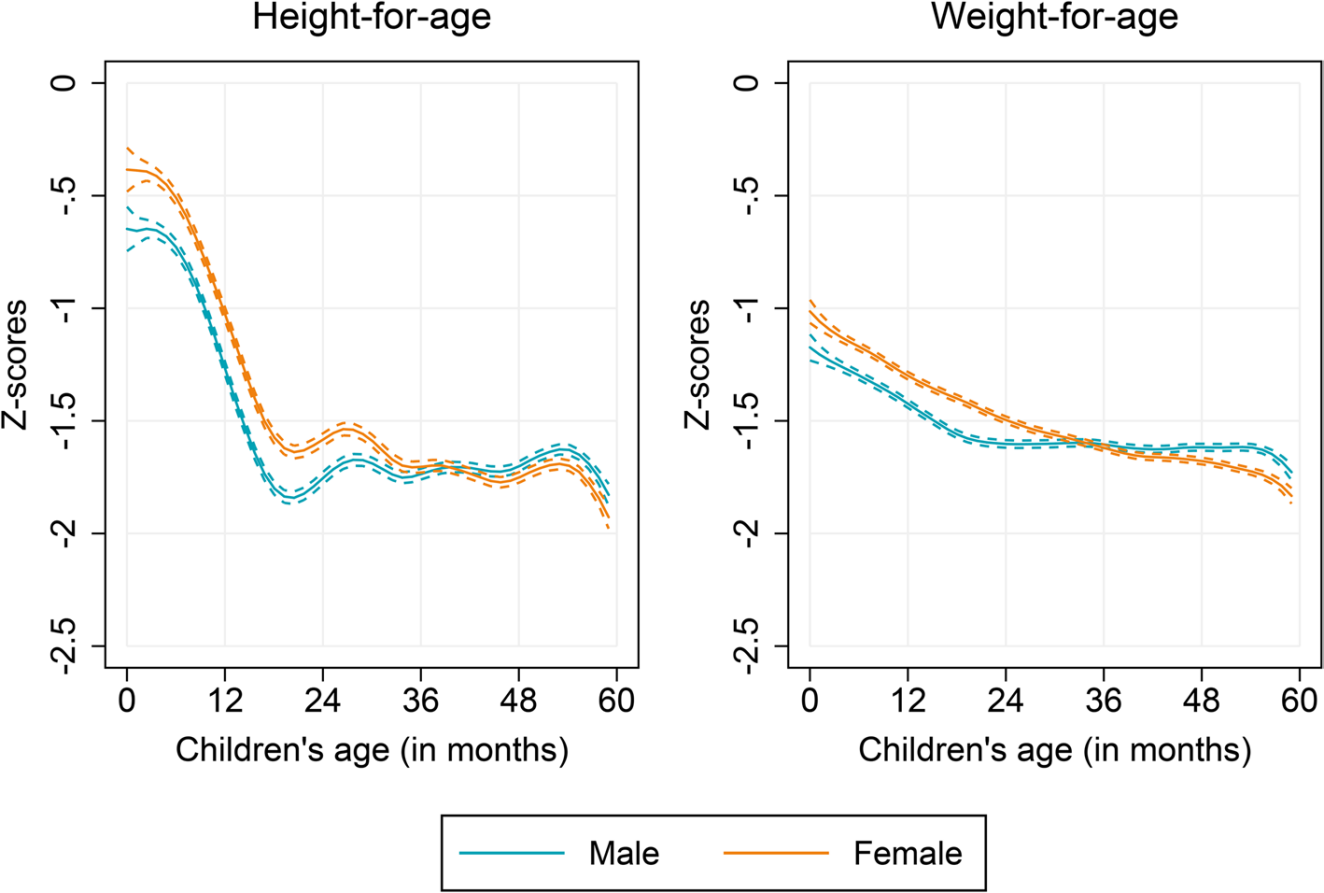
Anthropometric age profiles of under-five children by sex, India, 2015–16

**Fig. 3:**
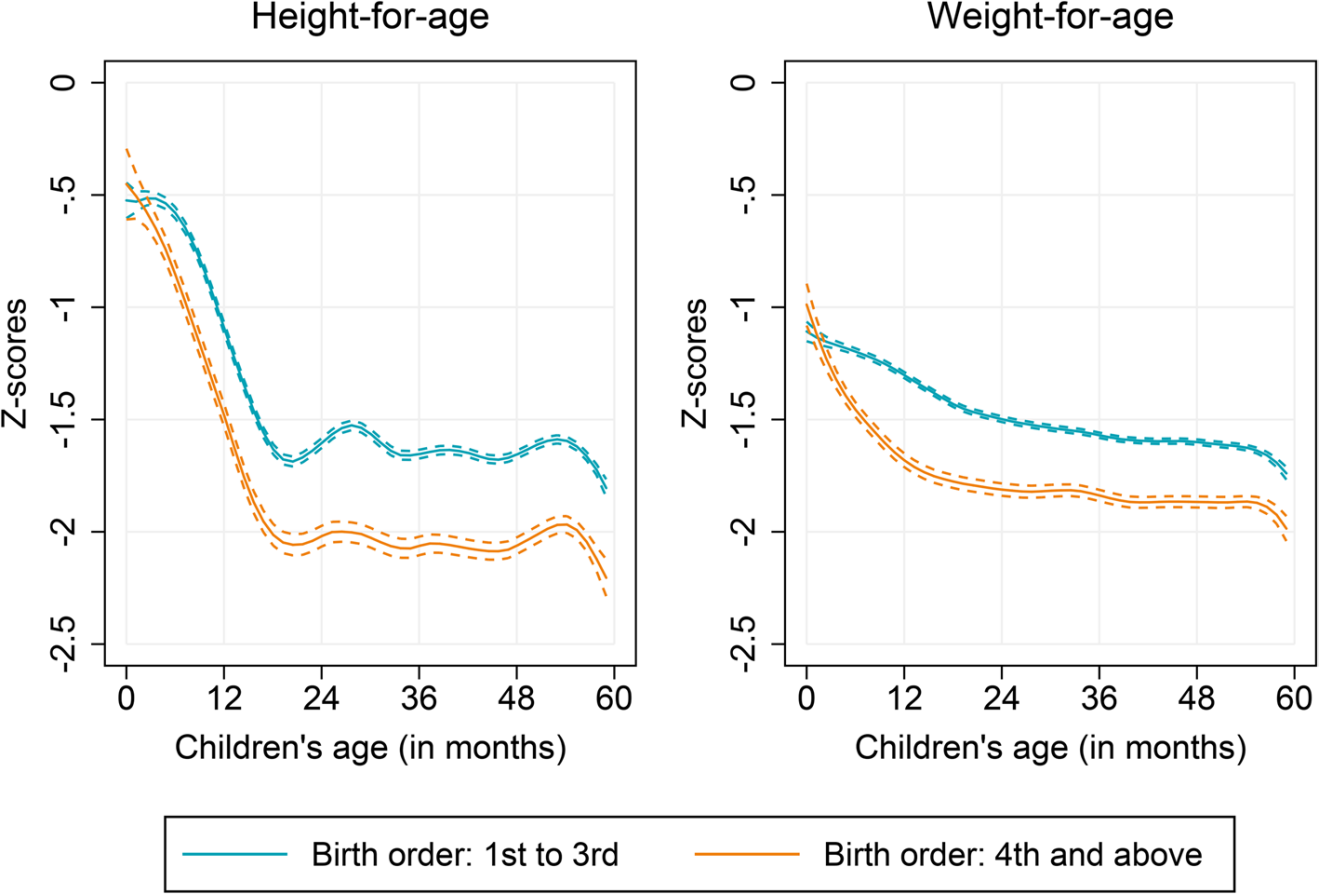
Anthropometric age profiles of under-five children by birth order, India, 2015–16

**Fig. 4:**
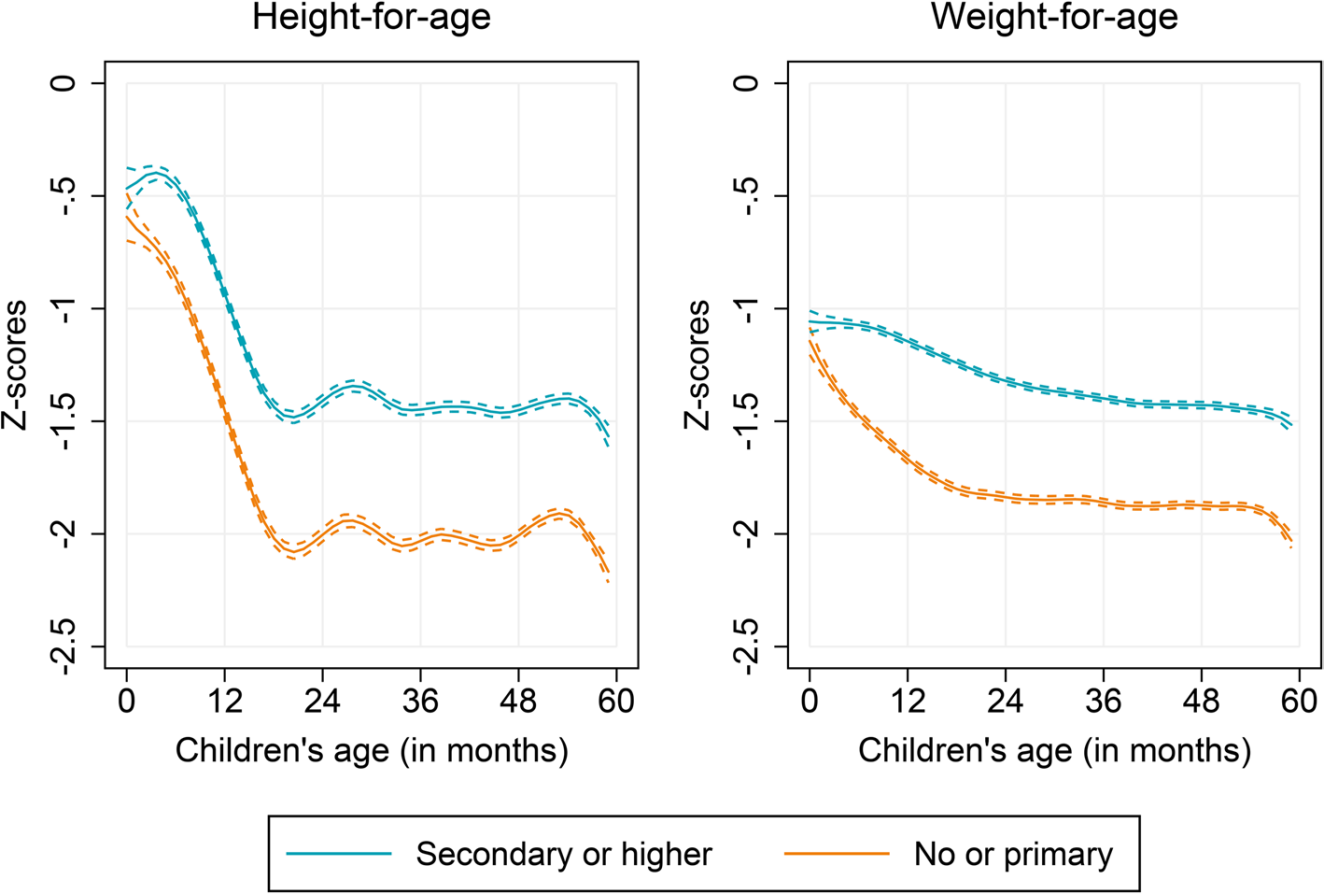
Anthropometric age profiles of under-five children by mother’s educational attainment, India, 2015–16

In order to document age heterogeneities in correlates of child growth, we begin by comparing the anthropometric age-profiles by several potential determinants of child growth, including the characteristics of child, mother, and household. We find no significant differences in (mean) HAZ and WAZ between male and female children over ages (Fig. 2). However, we notice substantial differences by age when children of lower birth order (first to third) are compared to that of higher birth orders (fourth and above) (Fig. 3). Children of higher birth orders lag behind those of lower birth orders over age, but the growth differences appeared more pronounced in older children. Similar to birth order, the preceding birth interval of children revealed important age heterogeneities (see Appendix Figure A1). Children who were born following a shorter preceding birth interval (PBI, less than 24 months) were considerably worse off among the older age groups. Furthermore, we find that children whose mothers completed at least secondary schooling were taller and heavier than children whose mothers only completed primary schooling or never attended school, and these differences were significantly larger in older children (Fig. 4). Similar to maternal education, we find striking age heterogeneities by household wealth status and household’s cooking fuel type (see Appendix Figures A2 and A3). Children living in poor households and in households those use solid cooking fuel are found to be both shorter and lighter, and this retardation in child’s growth appeared stronger with child’s age.

### Results from regression models

#### Height-for-age results

Table 2 summarises the results for HAZ, estimated from the four-level multilevel model. In the right panel, we present adjusted coefficients controlled for independent variables. The fourth column shows the main association between the independent variable and HAZ. The fifth column reports the interactive effects between each covariates and child’s age. Finally, results listed in the sixth column are the interactive effects between each independent variable and structural break. In the left panel, we present the unadjusted coefficients, explaining the independent associations of HAZ with each of the control variables and interaction terms.

**Table 2:** Estimates from multilevel models of height-for-age z-scores

Variables	Unadjusted Model			Adjusted Model		
	**Main effect**	**Interaction age**	**Interaction break**	**Main effect**	**Interaction age**	**Interaction break**
	**(1)**	**(2)**	**(3)**	**(4)**	**(5)**	**(6)**
Child's age	-0.018*	-	-	-0.112*	-	-
	(0.000)	-	-	(0.007)	-	-
Break	-0.586*	-	-	-1.890*	-	-
	(0.007)	-	-	(0.156)	-	-
Male	-0.248*	0.002	-0.073^**#**^	-0.259*	0.002	-0.062
	(0.023)	(0.002)	(0.041)	(0.023)	(0.002)	(0.040)
Higher birth order (4 +)	0.060	-0.019*	-0.411*	-0.056	-0.005^**#**^	-0.054
	(0.042)	(0.003)	(0.072)	(0.044)	(0.003)	(0.076)
Short preceding birth interval (< 24 months)	-0.102*	-0.011*	-0.409*	-0.094*	-0.008*	-0.318*
	(0.032)	(0.002)	(0.054)	(0.032)	(0.002)	(0.055)
Small birth size	-0.613*	0.020*	0.079*	-0.590*	0.022*	0.147*
	(0.035)	(0.003)	(0.001)	(0.035)	(0.003)	(0.063)
Mother's age at marriage	0.004	0.002*	0.050*	-0.004	0.001*	0.023*
	(0.003)	(0.000)	(0.005)	(0.003)	(0.000)	(0.006)
Mother's higher education	0.224*	0.018*	0.430*	0.106*	0.005^**#**^	0.133*
	(0.039)	(0.003)	(0.070)	(0.041)	(0.003)	(0.075)
Mother's height (in 10 cm)	0.239*	0.005*	0.170*	0.244*	0.004*	0.147*
	(0.012)	(0.001)	(0.020)	(0.012)	(0.001)	(0.021)
Mother's BMI (in kg/m^2^)	0.144*	0.002*	0.070*	0.143*	-0.001	-0.012
	(0.012)	(0.001)	(0.020)	(0.013)	(0.001)	(0.022)
Mother's media exposure	0.082*	0.016*	0.366*	0.007	0.005*	0.080^**#**^
	(0.024)	(0.002)	(0.041)	(0.029)	(0.002)	(0.051)
Solid fuel	-0.231*	-0.013*	-0.280*	-0.081*	0.002	0.059
	(0.026)	(0.002)	(0.044)	(0.026)	(0.002)	(0.045)
Poor wealth	-0.223*	-0.017*	-0.472*	-0.083*	-0.007*	-0.216*
	(0.023)	(0.002)	(0.040)	(0.034)	(0.003)	(0.059)
Urban residence	0.025	0.016*	0.279*	0.189*	0.011*	0.133*
	(0.029)	(0.002)	(0.047)	(0.035)	(0.003)	(0.059)
Proportion of poor HH in community^a^	-0.043*	-0.002*	-0.053*	-0.003	-0.001^**#**^	-0.023*
	(0.004)	(0.000)	(0.006)	(0.007)	(0.000)	(0.011)
Proportion of HH practices OD in community^a^	-0.049*	-0.001*	-0.041*	-0.027*	-0.001*	-0.004
	(0.003)	(0.000)	(0.005)	(0.005)	(0.000)	(0.008)
Proportion of mother not having primary schooling^a^	-0.029*	-0.003*	-0.071*	-0.002	-0.001*	-0.027*
	(0.004)	(0.000)	(0.007)	(0.005)	(0.000)	(0.009)
Proportion of children vaccinated in the district^a^	0.047*	0.006*	0.088*	0.079*	0.004*	0.058*
	(0.011)	(0.001)	(0.012)	(0.001)	(0.000)	(0.001)
Under-five mortality in the district (per 100 births)	-0.068*	-0.002*	-0.094*	-0.027*	< -0.001	-0.041*
	(0.007)	(0.000)	(0.009)	(0.007)	(0.000)	(0.010)
Constant	-	-	-	0.863*	-	-
	-	-	-	(0.097)	-	-
Variance: mother	-	-	-	0.441*	-	-
	-	-	-	(0.011)	-	-
Variance: community	-	-	-	0.141*	-	-
	-	-	-	(0.004)	-	-
Variance: district	-	-	-	0.042*	-	-
	-	-	-	(0.003)	-	-

Standard errors are clustered at the PSU level and are shown in parentheses

Interactions between variable and ‘age × break’ (*β*_*k‴*_) were estimated in both unadjusted as well as adjusted models, but are not shown

^a^ $\beta$ and SE are given for 10 percentage point change in the variable

^*^ *p* < 0.05, ^**#**^ *p* < 0.10

In bivariate analysis (unadjusted), each of the independent variables was significantly associated with HAZ (Table 2, column 1) and exhibited statistically significant (*P* < *0.05*) interaction terms on HAZ (Table 2, column 2 & 3). While the results from univariate analysis are important and have been crucial for electing the final set of covariates, we base our interpretation and finding as well as discussion for the adjusted estimates only.

From applied multilevel model, after controlling for a range of covariates and their interaction terms, we find many variables showing significant associations of considerable size. As shown in the fifth column of Table 2, being a male child, short preceding birth interval (PBI, < 24 months), small birth size, household’s poor wealth status, its usage of solid fuel, open defecation prevalence at community level, and under-five mortality at district level rate are negatively associated with HAZ. On the other hand, mother’s higher education, her height, BMI, and district-level immunization coverage rate are positively associated with HAZ.

We now turn to our main findings of interest, the heterogeneous effects of variables by child’s age. As presented in column 5 of Table 2, the significant negative coefficient on the interaction between child’s age and preceding birth interval suggests that moving from its value ≥ 24 months to < 24 months is associated with a sizable amount of HAZ decline for each month of age increase, leading to a substantial decline in HAZ during the first 23 months.^1^ Moreover, because of this strong steep decline during the first 23 months, the magnitude of faltering in HAZ gets substantially amplified among the children of older age group (i.e., 24–59 months, after the structural break) (see column 6 of Table 2). Interestingly, the coefficient of the interaction term between small birth size and child age is positive and statistically significant, suggesting that the negative effects of small birth size decreases with age. It is possible that children who born with small birth size, compared to those who born with average or larger than average birth size, are likely to gain more linear growth in height during the first 23 months. However, this benefit for linear growth in height, among those who born with small birth size, appears attenuated considerably in older age groups (24–59 months), as indicated by the coefficient (0.147, *P value* < 0.05) for the interaction between structural break and small birth size. The net effect of small birth size for height gain remains negative.

Regarding maternal characteristics, our findings suggest that the importance of mother’s education and her exposure to media on child’s height growth increases with child’s age. Children of better-educated mothers, that is, those who completed at least secondary schooling, are likely to gain their height progressively during the first two years and this progression seem to be more prominent among older children. The effect of having mother’s media exposure on child’s height growth is quantitatively very much similar to the effect of having hers better educational attainment. Similar to maternal education, the effect size of maternal height appeared to be more pronounced in children older than 2 years of age (i.e., after the structural break).

With respect to household characteristics, the interaction term between HAZ and poor wealth is significant and large in size, indicating that negative association between height gain and poor wealth tends to get stronger with child’s age. This reflects the sharp decline in HAZ during the first 23 months among children from poor households (i.e., bottom two wealth quintiles). Importantly, the scourges of lower wealth status are worse for children older than 2 years, as evidenced by the significant negative interaction term between structural break and lower wealth status.

Apart from child- and mother-level characteristics, community-level variables also exhibited differential effects by child’s age. Importantly, the associations of most community variables with height gain were small and more or less steady. The significant negative coefficient for the structural break, though small, implies depreciation in height gain among older children.

We also scrutinize that a positive association of district level childhood vaccination coverage with HAZ assures height gain in the children of younger age group of < 24 months. This association, however, attenuates significantly in children of 24–59 age groups, implying that the benefits of childhood vaccination are greater for children below 2 years of age.

The bottom part of Table 2 shows the variance estimates for HAZ, obtained from our adjusted model, suggesting that variation within mothers accounted for the highest proportion of the total variation in HAZ, followed by communities and districts.

#### Weight-for-age results

The adjusted coefficients (main effects) in column 4 of Table 3 suggest that weight-for-age z score (WAZ) is negatively associated with being a male child, higher birth order, short birth interval, and small birth size, household’s poor wealth status, living in a community with higher open defecation, higher maternal illiteracy, and living in a district with greater under-five mortality rate. Whereas, WAZ outcomes appeared to be better among the children who were born to taller mothers, mothers with greater BMI, and residing in an urban locality.

**Table 3:** Estimates from multilevel models of weight-for-age z-scores

Variables	Unadjusted Results			Adjusted Results		
	**Main effect**	**Interaction age**	**Interaction age-squared**	**Main effect**	**Interaction age**	**Interaction age-squared**
	**(1)**	**(2)**	**(3)**	**(4)**	**(5)**	**(6)**
Child's age	-0.008*	-		-0.062*	-	
	(0.000)	-		(0.011)	-	
Age squared	0.000*	-		0.001*	-	
	(0.000)	-		(0.000)	-	
Male	-0.104*	-0.009*	0.0005*	-0.108*	-0.008*	0.0003*
	(0.021)	(0.003)	(0.0001)	(0.020)	(0.003)	(0.0001)
Higher birth order (4 +)	0.034	-0.038*	0.0013*	-0.064^**#**^	-0.026*	0.0005*
	(0.037)	(0.005)	(0.0002)	(0.039)	(0.005)	(0.0002)
Short preceding birth interval (< 24 months)	-0.072*	-0.020*	0.0006*	-0.067*	-0.012*	0.0003*
	(0.028)	(0.004)	(0.0001)	(0.028)	(0.004)	(0.0001)
Small birth size	-0.612*	0.024*	-0.0006*	-0.588*	0.028*	-0.0005*
	(0.031)	(0.004)	(0.0002)	(0.031)	(0.004)	(0.0002)
Mother's age at marriage	0.001	0.003*	-0.0001*	-0.004	0.001*	< -0.0001^**#**^
	(0.003)	(0.000)	(0.0000)	(0.003)	(0.000)	(0.0000)
Mother's higher education	0.053*	0.041*	-0.0014*	-0.028	0.032*	-0.0006*
	(0.020)	(0.003)	(0.0001)	(0.027)	(0.005)	(0.0002)
Mother's height (in 10 cm)	0.172*	0.011*	-0.0003*	0.182*	0.007*	-0.0002*
	(0.010)	(0.001)	(0.0001)	(0.011)	(0.001)	(0.0001)
Mother's BMI (in kg/m^2^)	0.147*	0.012*	-0.0004*	0.151*	0.008*	-0.0002*
	(0.011)	(0.002)	(0.0001)	(0.012)	(0.002)	(0.0001)
Mother's media exposure	0.073*	0.033*	-0.0012*	0.040	0.004	-0.0001
	(0.021)	(0.003)	(0.0001)	(0.026)	(0.004)	(0.0001)
Solid fuel	-0.084*	-0.043*	0.0015*	-0.016	-0.006*	0.0002
	(0.023)	(0.003)	(0.0001)	(0.023)	(0.003)	(0.0001)
Poor wealth	-0.137*	-0.043*	0.0015*	-0.084*	-0.025*	0.0005*
	(0.021)	(0.003)	(0.0001)	(0.030)	(0.004)	(0.0002)
Urban residence	0.009	0.031*	-0.0010*	0.091*	0.002*	< -0.0001*
	(0.025)	(0.003)	(0.0001)	(0.031)	(0.004)	(0.0000)
Proportion of poor HH in community^a^	-0.026*	-0.006*	0.0002*	-0.031*	-0.003*	0.0001*
	(0.003)	(0.000)	(0.0000)	(0.006)	(0.001)	(0.0000)
Proportion of HH practices OD in community^a^	-0.048*	-0.002*	0.0001*	-0.040*	0.002	< -0.0001*
	(0.003)	(0.000)	(0.0000)	(0.004)	(0.001)	(0.0000)
Proportion of mother not having primary schooling^a^	-0.027*	-0.006*	0.0002*	-0.009*	-0.002*	0.0001*
	(0.004)	(0.000)	(0.0000)	(0.004)	(0.001)	(0.0000)
Proportion of children vaccinated in the district^a^	-0.039*	0.007*	-0.0002*	-0.084*	0.005*	-0.0001*
	(0.010)	(0.001)	(0.0000)	(0.007)	(0.001)	(0.0000)
Under-five mortality in the district	-0.082*	-0.003*	0.0001*	-0.051*	0.001	< -0.0001
	(0.007)	(0.001)	(0.0000)	(0.007)	(0.001)	(0.0000)
Constant	-	-	-	-0.085	-	-
	-	-	-	(0.088)	-	-
Variance: mother	-	-	-	0.319*	-	-
	-	-	-	(0.005)	-	-
Variance: community	-	-	-	0.058*	-	-
	-	-	-	(0.004)	-	-
Variance: district	-	-	-	0.056*	-	-
	-	-	-	(0.003)	-	-

Standard errors are clustered at the PSU level and are shown in parentheses

Interactions between variable and ‘age × age-squared’ (*β*_*k‴*_) were estimated in both unadjusted as well as adjusted models, but are not shown

^a^ $\beta$ and SE are given for 10 percentage point change in the variable

^*^p < 0.05, ^**#**^ *p* < 0.10

Similar to the association between HAZ and independent variables, age-varying effects of several correlates, operating at child, mother, community, and district levels, on the WAZ were large and significant. In terms of child’s gender, coefficients for both the age interactions, i.e., male × age and male × age-squared, were statistically significant (see columns 5 and 6 of Table 3) thereby indicating that the differential effect of gender by child’s age is non-linear. The significant negative coefficient of interaction between age and gender indicates that the decrease in WAZ values with age is greater among males than females; further, the significant positive coefficient of interaction between age-squared and gender suggests that the magnitude of WAZ decrease attenuates with age.^2^ A fairly similar pattern, with greater effect sizes in 0–23 months but not in older children, was found for higher birth order and short preceding birth interval. The negative (main) effect of short birth size reveals deleterious effect on WAZ; however, the coefficient of the interaction between short birth size and child’s age is positive and significant, and thereby implies that the deleterious effect of short birth size on WAZ reduces as children climb the age ladder.

With respect to maternal characteristics, we found four age interactions with mother’s age at marriage, mother’s higher education, height, and BMI were positive and significant; amongst them, the interaction effect was the largest for mother’s higher education. The main effect of mother’s characteristics on WAZ were positive and significant for height and BMI and non-significant for mother’s age at marriage and higher education. Given the main effects, the beneficial effects of these four maternal conditions are likely to increase with child age; however, these four maternal conditions interacted with age-squared showed large negative effect on WAZ. Hence, the net effect of maternal conditions was likely to reduce with child age.

The interaction between child’s age and poor wealth, i.e., the lower quintile of wealth index, appeared more pronounced in WAZ than it was in HAZ. While the interactions between age and poor wealth is negative, the interactions between age-squared and poor health is positive implying a steady value in WAZ in older children. Further, the proportion of poor households at community level showed sizable age effects, and the level of childhood vaccination coverage showed significant effect at the district level. At community level, the proportion of mothers not having primary schooling showed negative effect on WAZ in interaction with child's age.

Similar to HAZ, the variance estimates for WAZ were significant at the levels of mother, community, and district. The corresponding variance estimates were 0.32, 0.06, and 0.06, respectively (see the bottom part of Table 3).

#### Supplementary analyses

For robustness check, we conduct regression analysis of HAZ and WAZ for children 0–23 months and 24–59 months separately to investigate whether associations between growth indicators and their determinants strengthen or attenuate when using younger samples of children (i.e., 0–23 months) relative to older samples of children (i.e., 24–59 months). Of note, in the analysis of age-disaggregated samples, we employed the same set of variables as in our main analysis.

Table A1 (see Appendix) reports the results from HAZ regression for children 0–23 months and 24–59 months. When we compared the coefficients obtained from these two sub-samples, we find that the coefficients of many of the variables are much larger for children of 24–59 months relative to the sample of younger children. For example, the coefficients on maternal education and household wealth increased by 30–62% when switching from the 0–23 months to the 24–59 months sample. Results from WAZ regression for children 0-23 months and 24-59 months are reported in Appendix Table A2. Overall, the results obtained from the regression of two age-disaggregated samples strongly conform to that of our main analysis.

## Discussion

Studies of the determinants of child nutrition assess the associations of various socio-economic, bio-demographic, and environmental factors with anthropometric outcomes and do not explore differential effects of these variables by child’s age. In this study, we examined the differential effects by examining the interactions between socio-economic, demographic, and environmental determinants and child’s age with HAZ and WAZ among 203,533 children aged 0–59 months using multilevel models. Our findings ascertain that the interactions of child’s age with determinants of undernutrition remarkably explain the bend in child’s growth curve. The patterns of height and weight growth faltering vary across age groups of children. Further, our findings revealed a number of socio-economic conditions, at the levels of child, mother, and household, that contribute to the growth faltering process in under-five children in India. Overall, the findings of this paper contribute to the ongoing debate of the ‘*window of opportunity’* for child undernutrition by mainly capturing heterogeneous faltering pattern in child’s growth curve and identifying the age-specific roles of various socioeconomic variables in child growth.

The age heterogeneity in the process of growth faltering is modulated by a number of biological, maternal, household, and environmental conditions. For India, the factors such as short birth interval, higher birth order, poor wealth status lead to a substantial plunge in anthropometric age-profiles, thus presenting deleterious effects during the crucial period of *window of opportunity*, i.e. during the first two years. Whereas, higher education level of mother and her greater media exposure are the factors that may lead to revival or upward shift in child’s growth curve during this period. Nonetheless, the factors leading to revival or upward shift played a righteous role for faltering of child's growth curve through a significant contribution to heterogeneity in age profile. These findings build a consensus that the first two years of life constitute an significant period for preventing faltering that can reduce undernutrition rates. Importantly, results highlight that improvement in maternal, household conditions, and programme outcomes can contribute significantly at the different development stages including first two years of life.

A number of maternal, household conditions, and programme outcomes influence child’s growth curve strongly even after the second birthday, beyond the well adopted period of *window of opportunity* [44]. The finding emphasizes that children in these post two years of age are particularly vulnerable to further growth restriction. The findings further corroborate to the fact that the differentials in growth faltering post 2 years of age in children from different socioeconomic background are caused by poverty, food insecurity, and low living standard and not caused by the genetic and maternal factors. This needs to be recognized that while from birth to 2 years of age remains the salient period for targeting determinants of child growth faltering, there are strong scopes to revive growth faltering in children beyond two years of age.

It is worth discussing here why the effect size of covariates, such as mother’s higher education, media exposure, and vaccine coverage when interacted with child’s age gets amplified. In view of the cumulative advantage of health benefits, the findings suggest that the positive association between child’s age and these factors suggest a wide range of favourable circumstantial evidence for childcare (e.g., use of modern healthcare services, timely introduction of complementary feeding) that accrues at each point of a child’s growth, and thus, fosters child development [45]. Contrarily, children from poor households suffer not only from the wide variety of detrimental factors (e.g., poor living conditions, poor healthcare) that they encounter at any point of life, but also they are exposed to a greater vulnerabilities of environment and household factors that show deleterious effects over the course of their lives [29]. The cumulative effects of such detrimental factors cancels the expected development in child’s growth.

With respect to WAZ, our findings show that the effect of most socio-demographic risk factors on WAZ attenuates non-linearly with the child’s age. As a result, the association of these variables with WAZ reduces significantly in strength or sometimes gets disappeared in later childhood ages (approximately after 24–35 months). A statistical explanation regarding such effect attenuation would be that, unlike HAZ, there is less variation in WAZ within the categories of most growth correlates (e.g., poor vs. non-poor) in older children to explain. Another possible interpretation would be that, unlike HAZ, WAZ can get affected by short-term impacts of childhood diseases (e.g., diarrhoea, fever, acute respiratory infections) and change in dietary intake [46] and consequently, the cumulative health impacts of socio-demographic risk factors may not be appropriately portrayed by the WAZ in children.

### Study strengths

The present study has several key strengths. First, in order to model cross-sectional determinants of child growth, we adopted a multilevel modelling framework which allowed us to simultaneously account for both age interactions and the underlying hierarchical data structure and thus, greatly reducing estimation biases that are inherent in a dataset with hierarchical nature. Second, the dataset we used for the current analysis comes with a wealth of information about child nutrition and its associated bio-demographic, socioeconomic, environmental, and programmatic factors, thereby enabling us to investigate age heterogeneities across a wide range of covariates. Finally, our analysis is based on a sample of large enough size which allowed us to efficiently constructing the anthropometric age-profiles and introducing age interactions in our multilevel regression models.

### Study limitations

The present study does have a few limitations. First, we do not have nationally representative longitudinal data on Indian children’s anthropometric measures and thus, our analyses are based on cross-sectional data. Hence, the anthropometric age-profiles that we present in this paper are based on age-pooled data. Consequently, while interpreting the observed patterns in child growth, we have to assume that children of the younger age groups represent the previous position (nutritional) of children who are currently in older age groups and vice versa. Also note that the results interpreted above are solely associational and are not indicative of a causal relationship. Moreover, the use of cross-sectional data restricted us to investigate the track changes in the age-specific determinants over time. Second, in NFHS-4, we have data only for children aged 0–59 months and thus, we are unable to capture the growth pattern of children beyond these age limits. Third, the analysis presented in the current paper could be subject to sample selection bias resulting from the survey non-response on children’s height and weight biomarker measurement and the removal of children with implausible height and weight measures from our analytical sample. Fourth, the data on child’s birth size were based on mother’s self-report and therefore, could be subject to reporting bias. This is because, the perception of birth size, say, for example, small birth likely to vary from mother to mother as well from one region to other [47]. Finally, our choice of determinants affecting children’s growth is limited to what we avail from the survey data. As a result, several important determinants (e.g., nutritional intake of children, parental time allocation for child care, mother’s receiving of support from other family members, and frequency of childhood infections including diarrhoea, respiratory infections) could not be considered in the present analysis because they were not included or not measured systematically in the survey.

### Policy implications and future directions

Findings reported in this study have important implications for developing more focused nutrition and health interventions. For instance, the existence of strong positive age-wealth gradients in children’s growth outcomes suggests that interventions that target household economic aspects may have greater impacts on height growth in younger children. The impact of such interventions, however, appeared to diminish once the children grow older. Considering these age heterogeneities, interventions should be tailored with a more flexible approach, such as additional food and nutrition supplement programmes for age-specific poor-wealth groups, free provision of health counselling, etc., so that their impacts would be equivalent to that in younger children. Active initiatives should also be undertaken to reduce growth disparities resulting from maternal educational attainment. Second, persistent impacts of several variables even after the period of *window of opportunity* suggests that the effect of these variables should be considered in order to halt further growth restriction or improve child growth after two years of age.

Future research should consider longitudinal assessment of child growth patterns and age-varying impacts of socioeconomic and environmental factors on faltering child growth outcomes. Research is also needed to investigate how the dynamics of age moderates the associations between other measures of child’s health (e.g., haemoglobin levels, psychological outcomes) and socioeconomic correlates. Also needed is to disentangle the physio-biological pathways through which these age-varying associations function.

## Conclusions

In this study, we find that short birth interval, higher birth order, poor wealth status, higher under-five mortality rate at the district are associated with poor child growth. These factors are mainly responsible for a downward shift of growth curve during the first two years, i.e., the well-known period of *window of opportunity*. At the same time, the beneficial impacts of maternal conditions such as higher maternal education and greater height are found to accumulate during first two years. The net effect is a revival of the growth curve during window of opportunity. Further, the children born with short preceding birth intervals and those from poor households, in particular, need to be carefully followed up after 2 years of age, when these group of children are vulnerable to further growth restriction. The analyses explain the bend or faltering in the child’s age curve by socio-economic and demographic variables, and a larger contribution of underlying heterogeneities in older children. Overall, the findings of the present study provide evidence on the benefit of effective interventions aimed at preventing growth faltering at early ages.

## Supplementary Information

**Additional file 1: Fig. A1. **Anthropometric age profiles of under-five children by preceding birth interval (PBI), India, 2015-16. **Fig. A2. **Anthropometric age profiles of under-five children by household’s wealth status, India, 2015-16. **Fig. A3 **Anthropometric age profiles of under-five children by household’s type of fuel usage, India, 2015-16. **Table A1. **Estimates from split sample multilevel regression analysis of HAZ for children 0-23 months and 24-59 months. **Table A2. **Estimates from split sample multilevel regression analysis of WAZ for children 0-23 months and 24-59 months.

## Data Availability

The datasets analysed during the current study are available in the DHS Program repository, available upon request from: https://dhsprogram.com

## Footnotes

1 With a loss of 0.008 z-score for every 1-month age increase after birth, children with preceding birth interval ≥ 24 months, compared to children with preceding birth interval < 24 months, are likely to lose 0.184 (0.008 × 23 = 0.184) HAZ at the age of 23 months.

2 At the age of 1, 12, and 23 month(s), WAZ in males will be approximately -0.008, -0.053, and -0.025 unit, respectively, lower than the females of the same age groups. The following are the details for calculating the age-specific effect sizes of gender based on the estimates provided in Table 3 (columns 5 and 6): Effect size for a 1-month-old: (-0.008 × 1) + (0.0003 × 1^2^) = -0.0077. Effect size for a 12-month-old: (-0.008 × 12) + (0.0003 × 12^2^) = -0.0528. Effect size for a 23-month-old: (-0.008 × 23) + (0.0003 × 23^2^) = -0.0253.
