# Supplementary material for: Age heterogeneities in child growth and its associated socio-demographic factors: a cross-sectional study in India
Source: BMC Pediatr. 2022 Jun 30;22:384. doi: 10.1186/s12887-022-03415-x (PMC9248138; doi:10.1186/s12887-022-03415-x)
Supplement: Supplementary file 1 — Additional file 1: Fig. A1. Anthropometric age profiles of under-five children by preceding birth interval (PBI), India, 2015-16. Fig. A2. Anthropometric age profiles of under-five children by household’s wealth status, India, 2015-16. Fig. A3 Anthropometric age profiles of under-five children by household’s type of fuel usage, India, 2015-16. Table A1. Estimates from split sample multilevel regression analysis of HAZ for children 0-23 months and 24-59 months. Table A2. Estimates from split sample multilevel regression analysis of WAZ for children 0-23 months and 24-59 months. [file 12887_2022_3415_MOESM1_ESM.docx]

**Age heterogeneities in child growth and its associated socio-demographic factors: a cross-sectional study in India**

*Supplementary appendix*


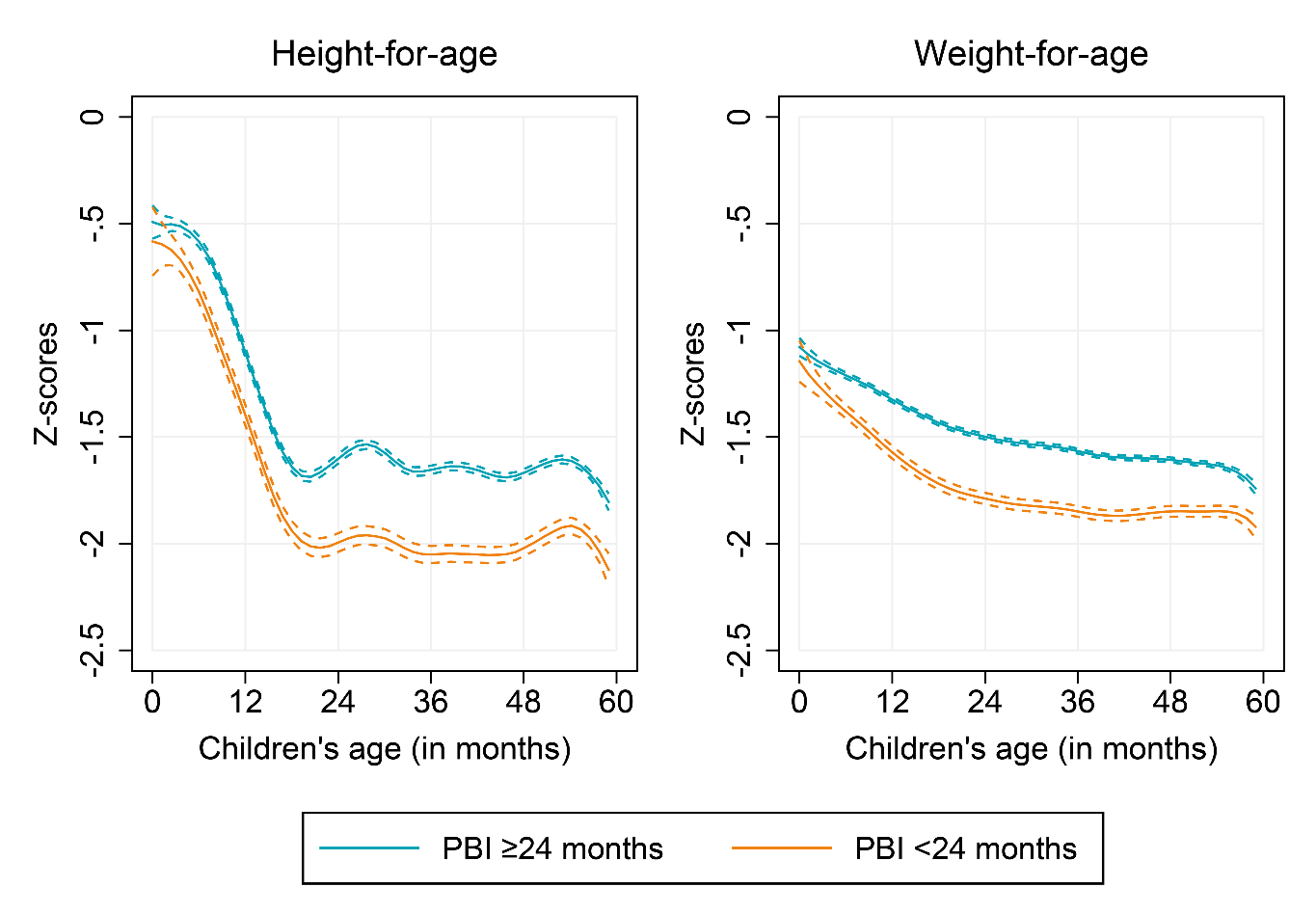


**Fig. A1** Anthropometric age profiles of under-five children by preceding birth interval (PBI), India, 2015-16


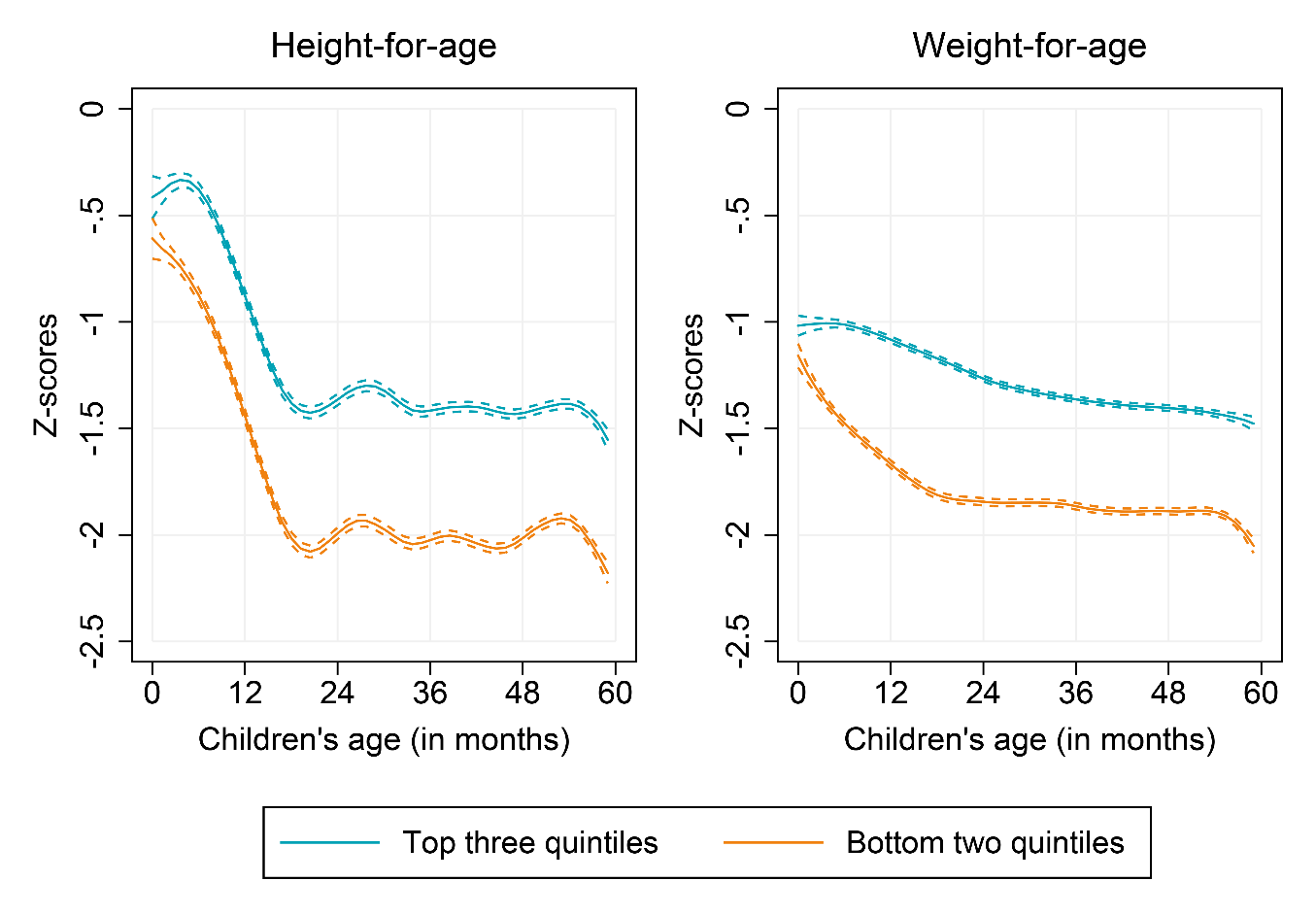


**Fig. A2** Anthropometric age profiles of under-five children by household’s wealth status, India, 2015-16


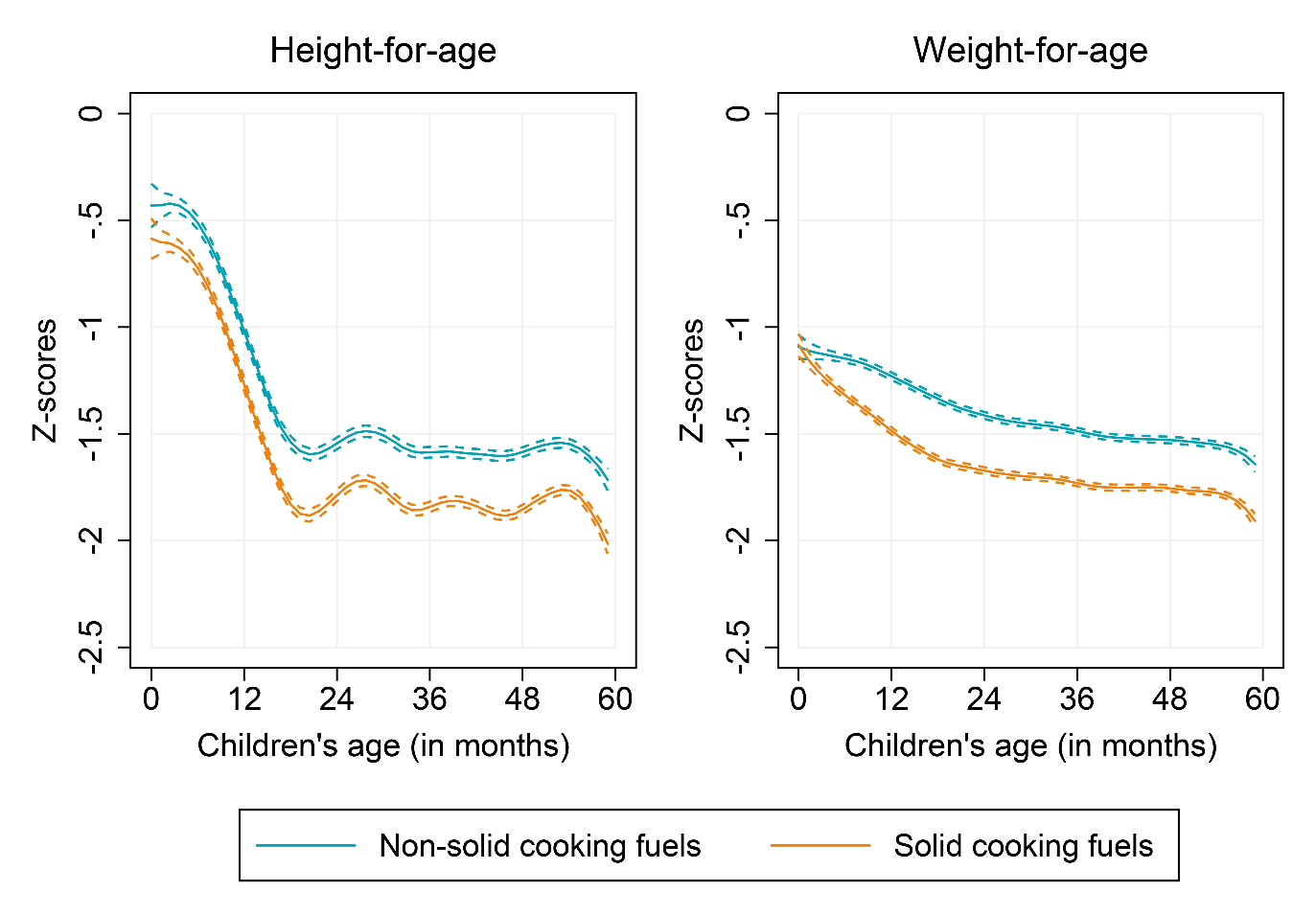


**Fig. A3** Anthropometric age profiles of under-five children by household’s type of fuel usage, India, 2015-16

**Table A1:** Estimates from split sample multilevel regression analysis of HAZ for children 0-23 months and 24-59 months

|  | **0-23 months** |  | **24-59 months** |
| --- | --- | --- | --- |
| **Variables** | **Coeff.**  **(Std. Err.)** |  | **Coeff.**  **(Std. Err.)** |
|  |  |  |  |
| Male | -0.219* |  | -0.017* |
|  | (0.012) |  | (0.008) |
|  |  |  |  |
| Higher birth order (4+) | -0.158* |  | -0.135* |
|  | (0.025) |  | (0.015) |
|  |  |  |  |
| Short preceding birth interval (<24 months) | -0.161* |  | -0.266* |
|  | (0.017) |  | (0.010) |
|  |  |  |  |
| Small birth size | -0.341* |  | -0.178* |
|  | (0.019) |  | (0.013) |
|  |  |  |  |
| Mother's age at marriage | 0.004**^#^** |  | 0.007* |
|  | (0.002) |  | (0.002) |
|  |  |  |  |
| Mother's higher education | 0.190* |  | 0.254* |
|  | (0.022) |  | (0.016) |
|  |  |  |  |
| Mother's height (in 10 cm) | 0.301* |  | 0.307* |
|  | (0.007) |  | (0.005) |
|  |  |  |  |
| Mother's BMI (in kg/m^2^) | 0.136* |  | 0.126* |
|  | (0.007) |  | (0.005) |
|  |  |  |  |
| Mother's media exposure | 0.066* |  | 0.089* |
|  | (0.016) |  | (0.011) |
|  |  |  |  |
| Solid fuel | -0.049* |  | -0.018**^#^** |
|  | (0.015) |  | (0.010) |
|  |  |  |  |
| Poor wealth | -0.166* |  | -0.196* |
|  | (0.018) |  | (0.012) |
|  |  |  |  |
| Urban residence | -0.058* |  | -0.017 |
|  | (0.022) |  | (0.015) |
|  |  |  |  |
| Proportion of poor HH in community^a^ | 0.003 |  | 0.003 |
|  | (0.005) |  | (0.004) |
|  |  |  |  |
| Proportion of HH practices OD in community^a^ | -0.017* |  | -0.012* |
|  | (0.004) |  | (0.003) |
|  |  |  |  |
| Proportion of mother not having primary schooling^a^ | -0.022* |  | -0.027* |
|  | (0.004) |  | (0.003) |
|  |  |  |  |
| Proportion of children vaccinated in the district^a^ | -0.026* |  | 0.006 |
|  | (0.007) |  | (0.006) |
|  |  |  |  |
| Under-five mortality in the district | -0.025* |  | -0.032* |
|  | (0.006) |  | (0.005) |
|  |  |  |  |
| Constant | 0.336* |  | -1.327* |
|  | (0.065) |  | (0.05) |
|  |  |  |  |
| Variance: mother | 0.823* |  | 0.529* |
|  | (0.056) |  | (0.015) |
|  |  |  |  |
| Variance: community | 0.260* |  | 0.166* |
|  | (0.011) |  | (0.006) |
|  |  |  |  |
| Variance: district | 0.063* |  | 0.052* |
|  | (0.006) |  | (0.004) |

**Table A2:** Estimates from split sample multilevel regression analysis of WAZ for children 0-23 months and 24-59 months

|  | **0-23 months** |  | **23-59 months** |
| --- | --- | --- | --- |
| **Variables** | **Coeff.**  **(Std. Err.)** |  | **Coeff.**  **(Std. Err.)** |
|  |  |  |  |
| Male | -0.126* |  | 0.016* |
|  | (0.009) |  | (0.006) |
|  |  |  |  |
| Higher birth order (4+) | -0.187* |  | -0.089* |
|  | (0.017) |  | (0.012) |
|  |  |  |  |
| Short preceding birth interval (<24 months) | -0.120* |  | -0.182* |
|  | (0.012) |  | (0.008) |
|  |  |  |  |
| Small birth size | -0.363* |  | -0.214* |
|  | (0.013) |  | (0.01) |
|  |  |  |  |
| Mother's age at marriage | 0.001 |  | 0.006* |
|  | (0.002) |  | (0.001) |
|  |  |  |  |
| Mother's higher education | 0.164* |  | 0.216* |
|  | (0.015) |  | (0.012) |
|  |  |  |  |
| Mother's height (in 10 cm) | 0.231* |  | 0.214* |
|  | (0.005) |  | (0.004) |
|  |  |  |  |
| Mother's BMI (in kg/m^2^) | 0.193* |  | 0.187* |
|  | (0.005) |  | (0.004) |
|  |  |  |  |
| Mother's media exposure | 0.063* |  | 0.058* |
|  | (0.011) |  | (0.008) |
|  |  |  |  |
| Solid fuel | -0.034* |  | -0.024* |
|  | (0.011) |  | (0.008) |
|  |  |  |  |
| Poor wealth | -0.164* |  | -0.148* |
|  | (0.012) |  | (0.009) |
|  |  |  |  |
| Urban residence | -0.079* |  | -0.055* |
|  | (0.014) |  | (0.011) |
|  |  |  |  |
| Proportion of poor HH in community^a^ | 0.004 |  | 0.003* |
|  | (0.004) |  | (0.003) |
|  |  |  |  |
| Proportion of HH practices OD in community^a^ | -0.021* |  | -0.014* |
|  | (0.003) |  | (0.003) |
|  |  |  |  |
| Proportion of mother not having primary schooling^a^ | -0.020* |  | -0.018* |
|  | (0.003) |  | (0.002) |
|  |  |  |  |
| Proportion of children vaccinated in the district^a^ | -0.043* |  | -0.018* |
|  | (0.006) |  | (0.005) |
|  |  |  |  |
| Under-five mortality in the district | -0.034* |  | -0.022* |
|  | (0.006) |  | (0.005) |
|  |  |  |  |
| Constant | -0.416* |  | -1.217* |
|  | (0.052) |  | (0.043) |
|  |  |  |  |
| Variance: mother | 0.403* |  | 0.352* |
|  | (0.025) |  | (0.009) |
|  |  |  |  |
| Variance: community | 0.062* |  | 0.069* |
|  | (0.005) |  | (0.003) |
|  |  |  |  |
| Variance: district | 0.069* |  | 0.053* |
|  | (0.005) |  | (0.004) |
